# Impact of clothianidin exposure on the growth, metabolism, and neurological function of *Penaeus vannamei*

**DOI:** 10.1007/s44154-025-00259-0

**Published:** 2025-11-02

**Authors:** Zhi Luo, Zhen-Fei Li, Zhi-Yu Lin, Zhen-Qiang Fu, Feng-Lu Han, Er-Chao Li

**Affiliations:** 1 School of Life Sciences, East China Normal University, 500 Dongchuan Road, Shanghai, 200241 China; 2 School of Marine Biology and Fisheries, Hainan University, Haikou, Hainan, 570228 China; 3 School of Marine Science, Sun Yat-Sen University, Zhuhai, Guangdong, 519082 China

**Keywords:** Neonicotinoid insecticides, Toxicity, Shrimp, Mechanism

## Abstract

Clothianidin, a widely used neonicotinoid pesticide, poses potential ecological risks to aquatic ecosystems due to its unique mode of action and widespread environmental dispersal. This study investigates the toxic effects of clothianidin on *Penaeus vannamei* at different concentrations over 28 days. High concentrations of clothianidin significantly affected shrimp physiology, as evidenced by changes in survival rate and weight gain. Markers of oxidative stress, including decreased respiratory burst, reduced glutathione levels, and diminished antioxidant enzyme activities, indicated that clothianidin triggered oxidative stress responses in shrimp. Additionally, changes in lactate dehydrogenase, succinate dehydrogenase, and isocitrate dehydrogenase activities suggested disruptions in energy metabolism in the hepatopancreas. Analysis of the nervous system revealed significant disturbances in neural signaling, reflected by altered levels of acetylcholine, acetylcholinesterase, and dopamine. Transcriptomic analysis highlighted significant changes in gene expression and metabolic processes in the nervous system. This study demonstrates that clothianidin disrupts oxidative balance, energy metabolism, and neural signaling, affecting the growth of *P. vannamei* and providing valuable insights into its biochemical and transcriptomic toxicity in aquatic environments.

**Graphic Abstract:**

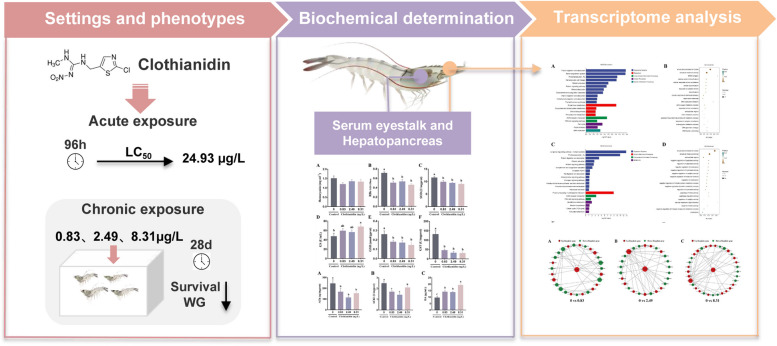

**Supplementary Information:**

The online version contains supplementary material available at 10.1007/s44154-025-00259-0.

## Introduction

Since the mid-1940s, with the development of commercial agriculture, the global demand for pesticides has increased sharply and steadily (Ahmad et al. 2024). However, the widespread use of chemical pesticides in agriculture has placed immense pressure on human health, biodiversity, and the environment (Cech et al. 2023). Neonicotinoid insecticides have become the most widely used class of insecticides globally, accounting for 26% of the global insecticide market (Miles et al. 2017). As one of the most commonly used neonicotinoids, clothianidin, due to its long half-life, high water solubility, and leaching potential, has a high detection rate in surface water, leading to its transport to aquatic environments (Morrissey et al. 2015). Environmental concentrations of clothianidin detected in aquatic environments range from < 0.025 to 3.5 μg/L, which are equal to or exceed the acute and chronic exposure threshold concentrations for many common aquatic invertebrate species (Main et al. 2014; 2017; Wang et al. 2023). It has been reported that clothianidin is bioaccumulated by various aquatic organisms, from plankton to higher trophic organisms, thereby promoting its accumulation in the food chain and ultimately impacting human health through seafood consumption (Malhotra et al. 2021).

In recent years, numerous studies have explored the toxicity of neonicotinoid insecticides on non-target organisms. For example, clothianidin has been shown to induce inflammatory responses and affect the reproductive system in rats (Onaru et al. 2020; Bal et al. 2013). It also weakens the immune system in bees, disrupts foraging behavior, and reduces population numbers (Prisco et al. 2013; Morfin et al. 2019; Yamada et al. 2012). Additionally, it affects the movement of the monarch butterfly (*Danaus plexippus*) (Pecenka and Lundgren (Pecenka and Lundgren 2015). Clothianidin has been found to reduce the feeding rate in *Belostoma flumineum* and lower the responsiveness of *Orconectes propinquus* (Miles et al. 2017). However, research on the toxicity of clothianidin to aquatic crustaceans remains limited. Existing studies indicate that clothianidin affects the behavior and physiological functions of aquatic invertebrates by disrupting their nervous systems, but the comprehensive impact on growth, metabolism, immunity, and gene expression at varying exposure concentrations has not been fully explored (Miles et al. 2017; Morrissey et al. 2015; Malhotra et al. 2021). Furthermore, the long-term accumulation of clothianidin in aquatic environments and its potential ecological impact remain unresolved scientific questions that require further investigation.

Shrimp species are an important source of animal protein and trace elements in human nutrition, particularly in developing countries (Butcherine et al. 2021). In 2022, the global aquaculture production of*P. vannamei* reached 6.8 million tons, making it the leading species and the second most traded aquatic product, highly favored by consumers (Naidu et al. 2025). However, the rapid increase of clothianidin in aquatic environments poses a significant threat to the sustainability and safety of shrimp farming (Hladik and Kolpin 2016). Neonicotinoid insecticides act as potent agonists on the nicotinic acetylcholine receptors (nAChRs) in the central nervous systems of many insect species, while exhibiting antagonistic effects on non-arthropod aquatic invertebrates (Tufi et al. 2015). Although these compounds primarily target nAChRs in the brains of insects, studies have shown that exposure to neonicotinoid pesticides can alter olfactory and odor coding as well as sensory information processing in insects, such as in honeybees (*Apis mellifera*), leading to impaired foraging, learning, and memory abilities (Andrione et al. 2016; Siviter et al. 2018). However, numerous studies have found that other arthropods, including crustaceans, are similarly affected due to their similar nervous system structure (Anderson et al. 2015).

To better understand how neonicotinoid compounds affect aquatic invertebrates, this study exposed juvenile *P. vannamei* to environmentally relevant concentrations of clothianidin and assessed toxicity-related indicators along with transcriptomic analyses. These findings systematically reveal the toxic effects of clothianidin on *P. vannamei*. The results will provide scientific evidence for environmental policies and sustainable aquaculture practices, making a significant contribution to environmental toxicology and the protection of aquatic ecosystems.

## Results

### Acute toxicity test

**Fig. 1:**
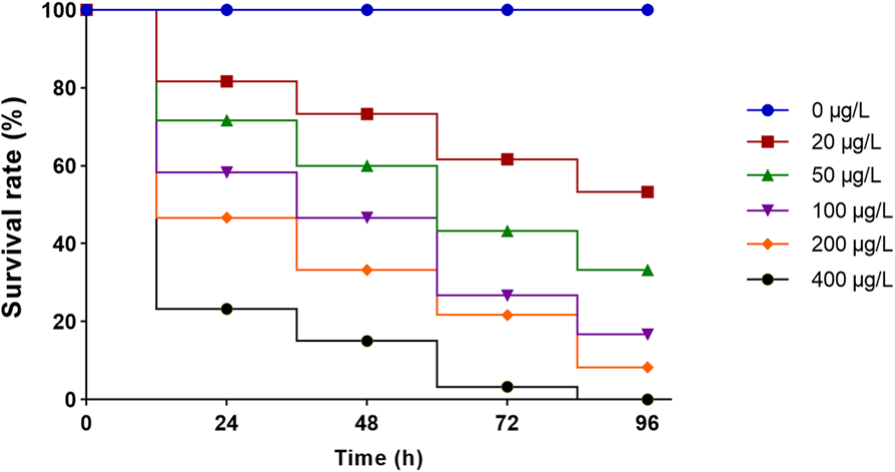
Response of the survival rate of *P. vannamei* to different concentrations of clothianidin

After the 96-h acute toxicity test, the survival of *P. vannamei* in each experimental group was rigorously counted. The survival rate of the control group was 100%, and the number of dead individuals increased significantly in a dose-dependent manner as the Clothianidin exposure concentration increased. Toxicological analysis revealed that the 96-h median lethal concentration (LC_50_) of Clothianidin for *P. vannamei* was 24.93 μg/L, with a 95% confidence interval ranging from 16.38 to 33.24 μg/L (Fig. 1).

### Growth performance

**Fig. 2:**
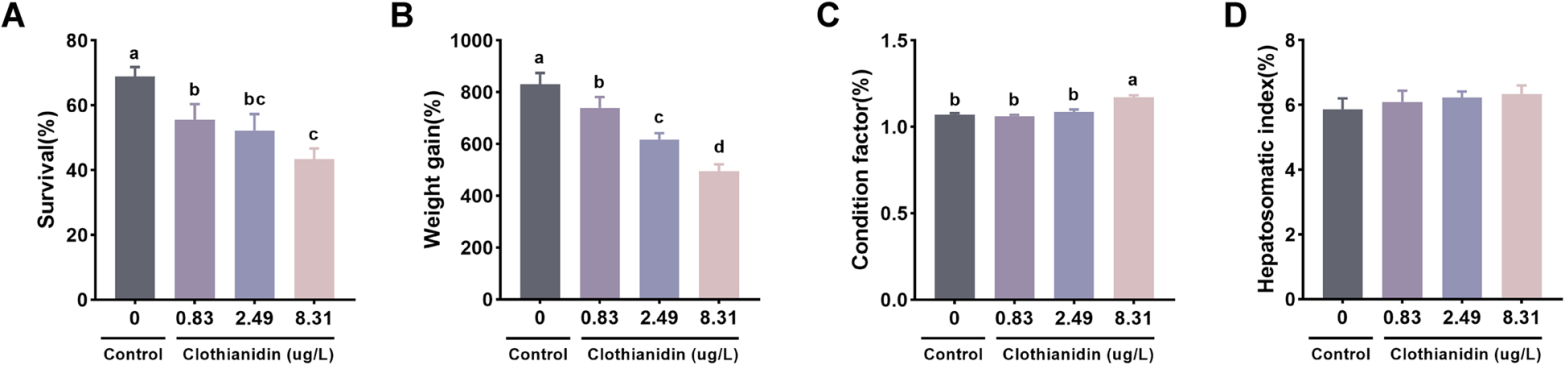
Growth performance of shrimp exposed to clothianidin for 28 days. **A** Survival; **B** weight gain; **C** condition factor; **D** hepatosomatic index. Error bars represent the mean ± SE (*n* = 4). Different letters represent significant differences between groups (*P* < 0.05)

As shown in Fig. 2, after 28 days of exposure, the survival rate and weight gain rate of the three exposure groups were significantly lower than those of the control group, with the 8.31 μg/L group showing the lowest survival and weight gain rates (*P* < 0.05, Fig. 2A, B). The condition factor of the 8.31 μg/L exposure group was significantly higher than that of the other groups (*P* < 0.05, Fig. 2C). No significant changes were observed in the hepatosomatic index (HSI) among all exposure groups (*P* > 0.05, Fig. 2D).

### Antioxidant capacity and detoxification metabolism

**Fig. 3:**
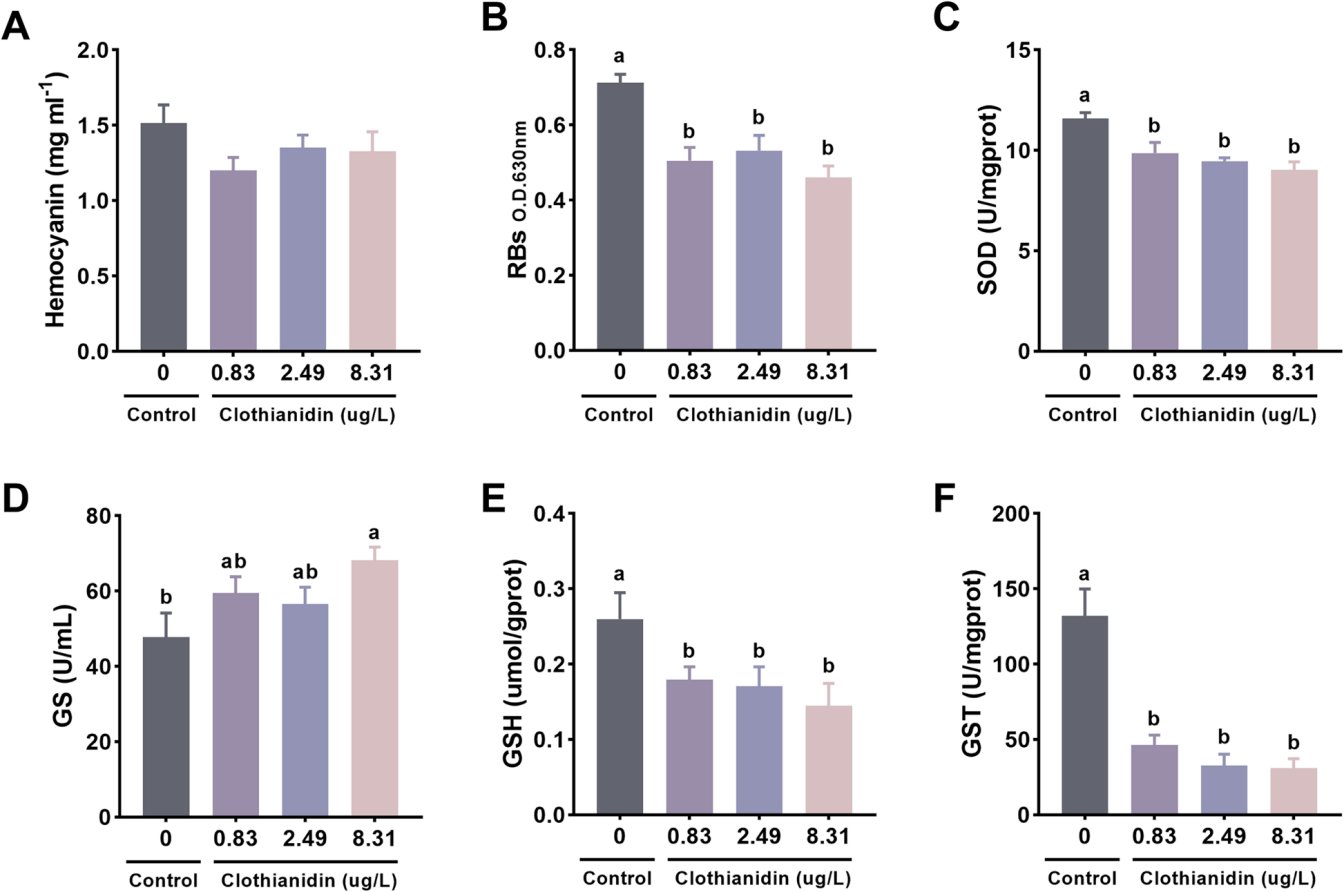
Effects of clothianidin on antioxidant capacity and detoxification metabolism in *P.vannamei* under chronic exposure. **A** Hemocyanin; **B** Respiratory burst (RBs); **C** Superoxide dismutase (SOD); **D** Glutamine synthetase (GS); **E** Reduced glutathione (GSH); **F** Glutathione S-transferase (GST). Different superscript letters denote statistically significant differences among groups (*P* < 0.05)

As shown in Fig. 3, no significant differences in hemocyanin levels were observed between the exposure groups and the control group (*P* > 0.05, Fig. 3A). Compared to the control group, the respiratory burst (RBs) levels of all three exposure groups were significantly lower (*P* < 0.05, Fig. 3B). In the hepatopancreas of the exposure groups, significant reductions in superoxide dismutase (SOD), reduced glutathione (GSH), and glutathione S-transferase (GST) levels were observed (*P* < 0.05, Fig. 3C, E, F). The glutamine synthetase (GS) content in the 8.31 μg/L exposure group was significantly higher than in the other groups (*P* < 0.05, Fig. 3D).

### Energy metabolism and signal regulation

**Fig. 4:**
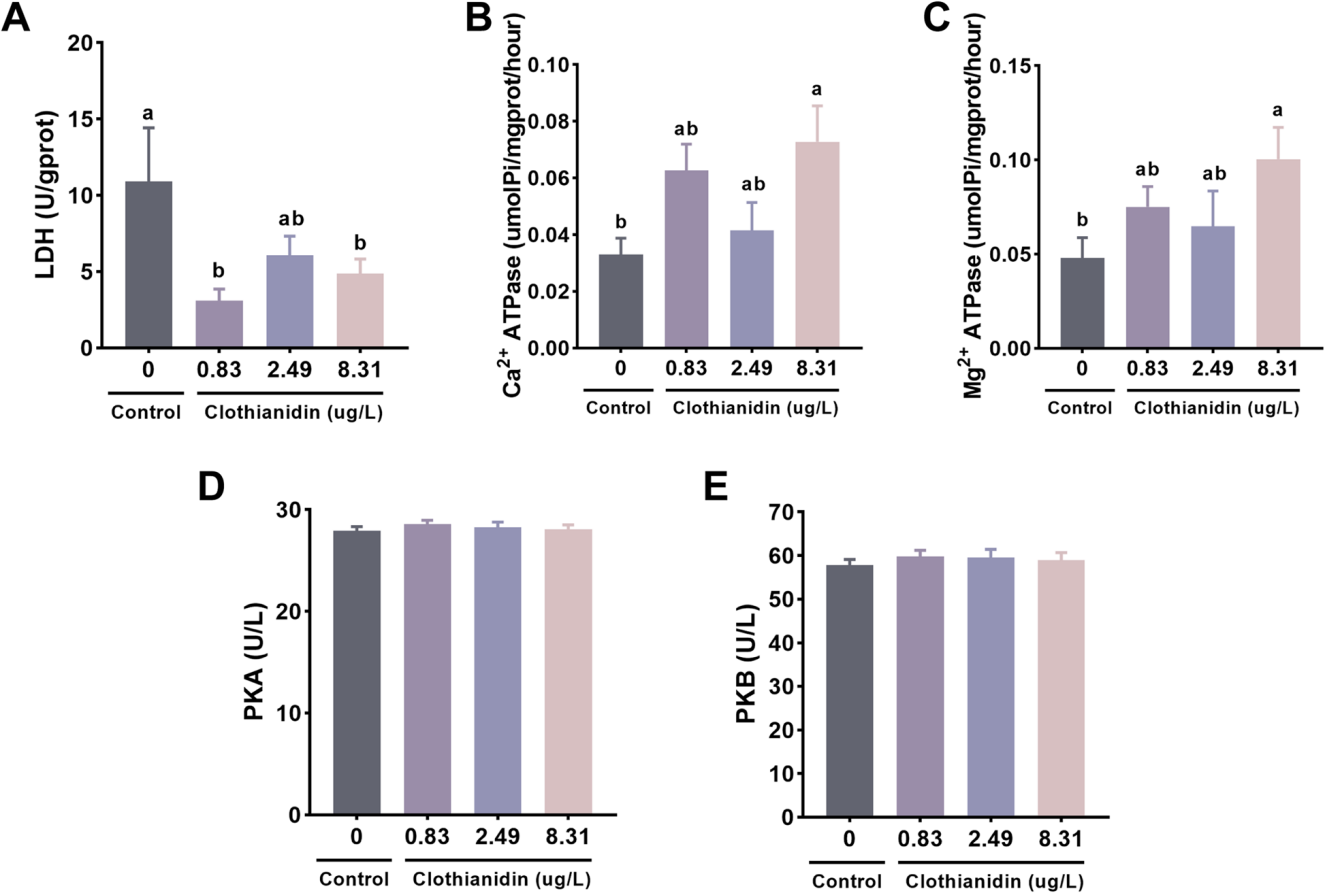
Effects of clothianidin on energy metabolism and signaling regulation in *P. vannamei* under chronic exposure. **A** Lactate dehydrogenase (LDH); **B** Ca^2^⁺-ATPase; **C** Mg^2^⁺-ATPase; **D** Protein kinase A (PKA); **E** Protein kinase B (PKB); **F** Glutathione S-transferase (GST). Different superscript letters indicate statistically significant differences among groups (*P* < 0.05)

Compared to the control group, the lactate dehydrogenase (LDH) levels in the 0.83 μg/L and 8.31 μg/L exposure groups were significantly lower (*P* < 0.05, Fig. 4A). The activity of Ca^2^⁺-ATPase and Mg^2^⁺-ATPase in the 8.31 μg/L exposure group was significantly higher than that in the control group (*P* < 0.05, Fig. 4B, C). No significant differences in PKA and PKB levels were observed between the exposure groups and the control group (*P* > 0.05, Fig. 4D, E).

### Neural signal transduction

**Fig. 5:**
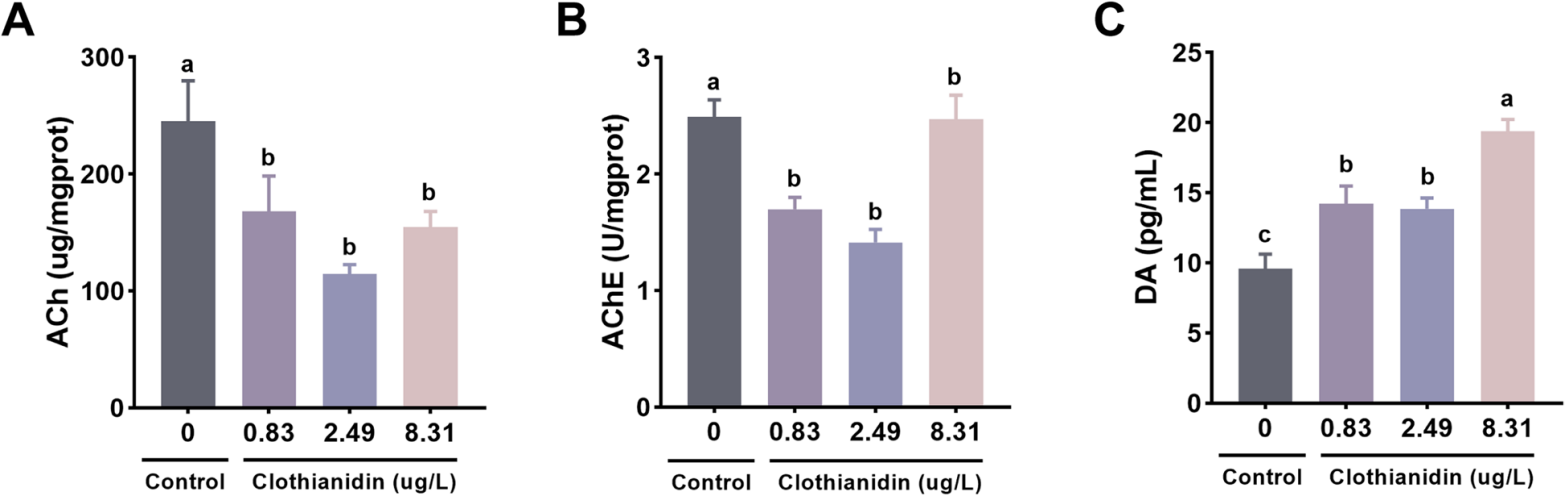
Effects of clothianidin on neurotransmission in *P. vannamei* under chronic exposure. **A** Acetylcholine (ACh); **B** Acetylcholinesterase (AChE); **C** Dopamine (DA). Different superscript letters indicate statistically significant differences among groups (*P* < 0.05)

Compared to the control group, the levels of acetylcholine (ACh) and acetylcholinesterase (AChE) in the three exposure groups were significantly lower (*P* < 0.05, Fig. 5A, B). The dopamine (DA) levels in all three exposure groups were significantly higher than those in the control group, with the 8.31 μg/L exposure group showing the highest dopamine (DA) levels (*P* < 0.05, Fig. 5C).

### Transcriptomics of the eyestalk

To elucidate the toxicological mechanisms of clothianidin exposure in *P. vannamei*, this study conducted transcriptome sequencing of the eyestalk tissue to analyze its molecular regulatory network. Quality control results of the sequencing data showed that a total of 575,655,520 raw reads were obtained from 12 samples. After quality filtering, each sample had more than 543,292,664 valid reads, with a total of 82,037,192,264 high-quality clean reads. Data quality assessment revealed that the Q20 and Q30 values for all samples were consistently above 97.92% and 94.26%, respectively. The reference genome alignment rate of the clean reads ranged from 94.32% to 94.49%.

**Fig. 6:**
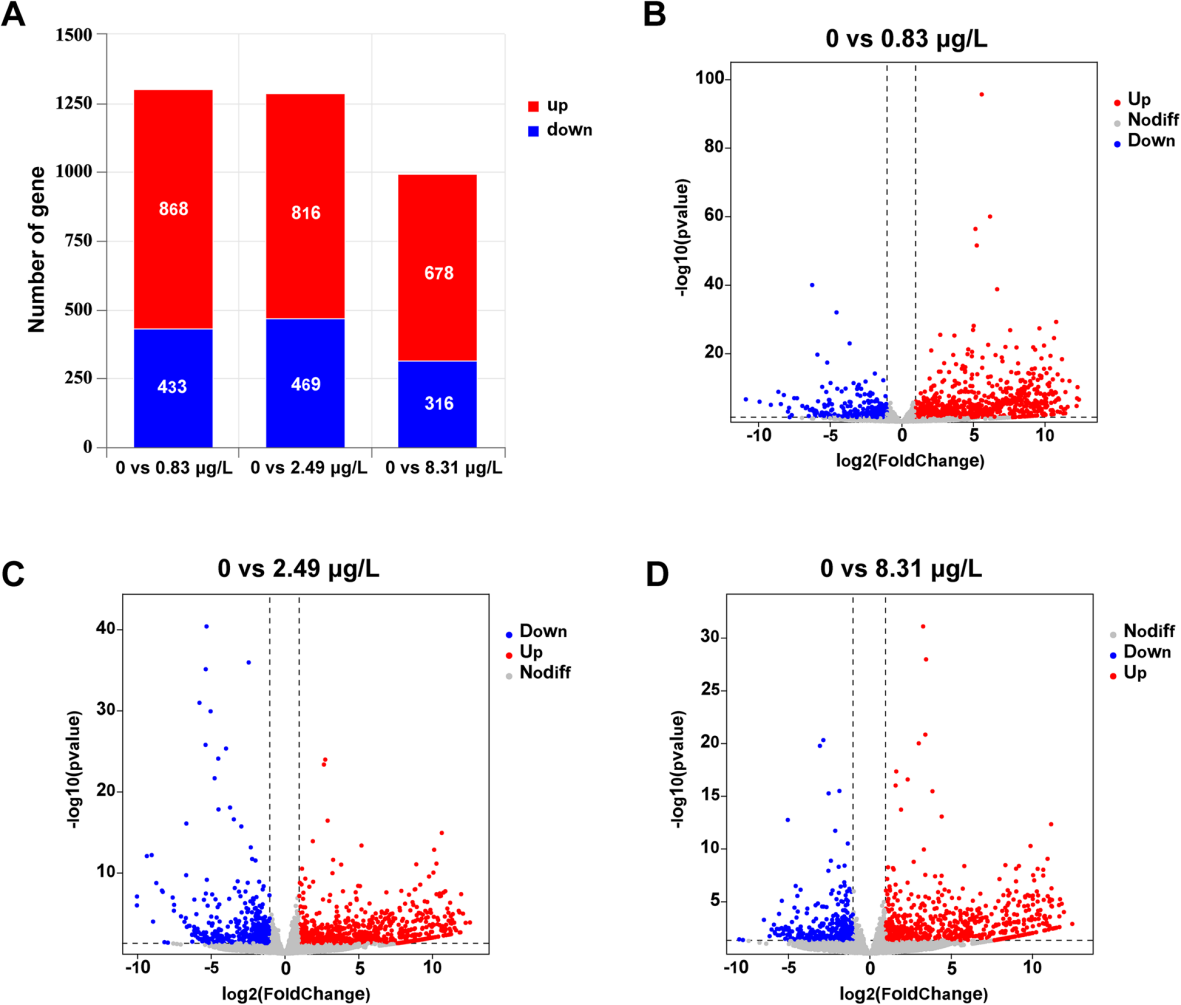
Transcriptomic changes in shrimp after 28 days of exposure to Clothianidin. **A** Proportional distribution of upregulated and downregulated genes in each comparison group. **B** Volcano plot of differentially expressed genes in the 0 vs 0.83 μg/L group. **C** Volcano plot of differentially expressed genes in the 0 vs 2.49 μg/L group. **D** Volcano plot of differentially expressed genes in the 0 vs 8.31 μg/L group

Transcriptomic results revealed significant effects of different concentrations of clothianidin on gene expression. The number of differentially expressed genes (DEGs) varied across the “0 vs 0.83 μg/L,” “0 vs 2.49 μg/L,” and “0 vs 8.31 μg/L” comparisons (Fig. 6A). Specifically, in the “0 vs 0.83 μg/L” group, 868 upregulated genes and 433 downregulated genes were detected; in the “0 vs 2.49 μg/L” group, there were 816 upregulated genes and 469 downregulated genes; and in the “0 vs 8.31 μg/L” group, 678 upregulated genes and 316 downregulated genes were identified (Fig. 6).

### KEGG and GO Enrichment analysis

**Fig. 7:**
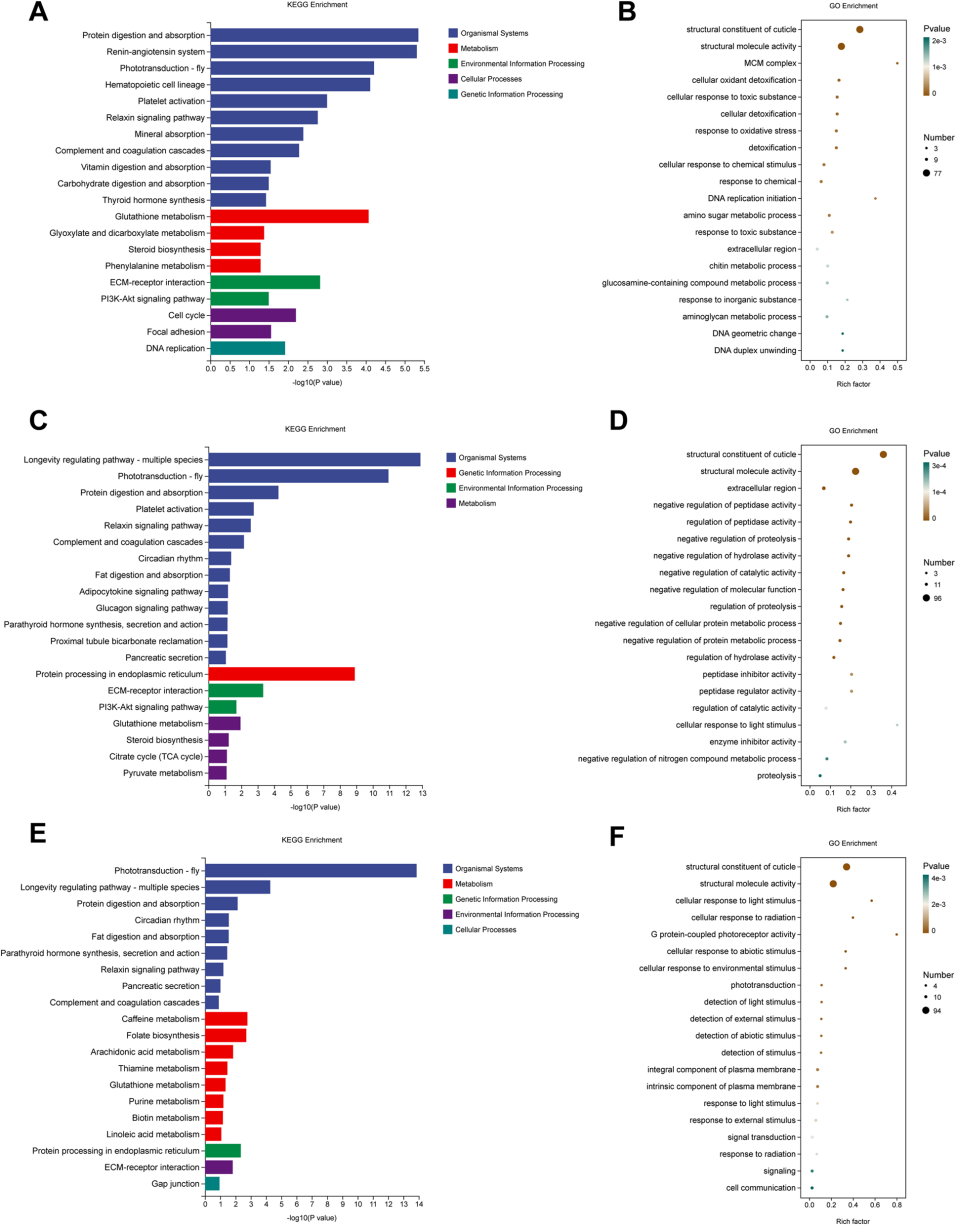
Transcriptomic changes in shrimp after 28 days of exposure to Clothianidin at different concentrations. **A** KEGG enrichment analysis for the 0 vs 0.83 μg/L group; **B** GO enrichment analysis for the 0 vs 0.83 μg/L group; **C** KEGG enrichment analysis for the 0 vs 2.49 μg/L group; **D** GO enrichment analysis for the 0 vs 2.49 μg/L group; **E** KEGG enrichment analysis for the 0 vs 8.31 μg/L group; **F** GO enrichment analysis for the 0 vs 8.31 μg/L group

In the “0 vs 0.83 μg/L” group, KEGG analysis revealed that multiple pathways were significantly enriched under clothianidin exposure, particularly those related to tissue systems, such as protein digestion and absorption, the angiotensin system, and phototransduction (fly). Among metabolic pathways, glutathione metabolism was the most significantly enriched, followed by pathways related to glyoxylate and dicarboxylate metabolism, steroid biosynthesis, and phenylalanine metabolism. GO enrichment analysis highlighted the structural components of the exoskeleton and structural molecular activity. Pathways related to toxin stress, including cellular oxidation detoxification, cellular response to toxic substances, and oxidative stress response, were also significantly enriched (Fig. 7A, B, Table S1, S2).

In the “0 vs 2.49 μg/L” group, KEGG analysis revealed multiple significantly enriched pathways, especially those related to longevity regulation (multi-species), phototransduction (fly), and protein digestion and absorption. Additionally, protein processing in the endoplasmic reticulum was also significantly enriched. Among metabolic pathways, glutathione metabolism, steroid biosynthesis, citric acid cycle (TCA cycle), and pyruvate metabolism were strongly enriched. GO enrichment analysis showed significant enrichment in protease activity, protein metabolic processes, and protein degradation. The structural components of the exoskeleton and structural molecular activity were also significantly enriched (Fig. 7C, D, Table S1, S2).

In the “0 vs 8.31 μg/L” group, KEGG analysis highlighted phototransduction (fly) and longevity regulation pathways (multi-species) as significantly enriched. Among metabolic pathways, caffeine metabolism, folate biosynthesis, and arachidonic acid metabolism were prominent in the enrichment analysis. Protein processing (endoplasmic reticulum) and ECM-receptor interaction also showed significant enrichment. GO enrichment analysis revealed significant enrichment in cellular response to light stimulus, response to environmental stimuli, and light receptor activity. Cellular communication and signal transduction pathways were also significantly enriched (Fig. 7E, F, Table S1, S2).

### GSEA

**Fig. 8:**
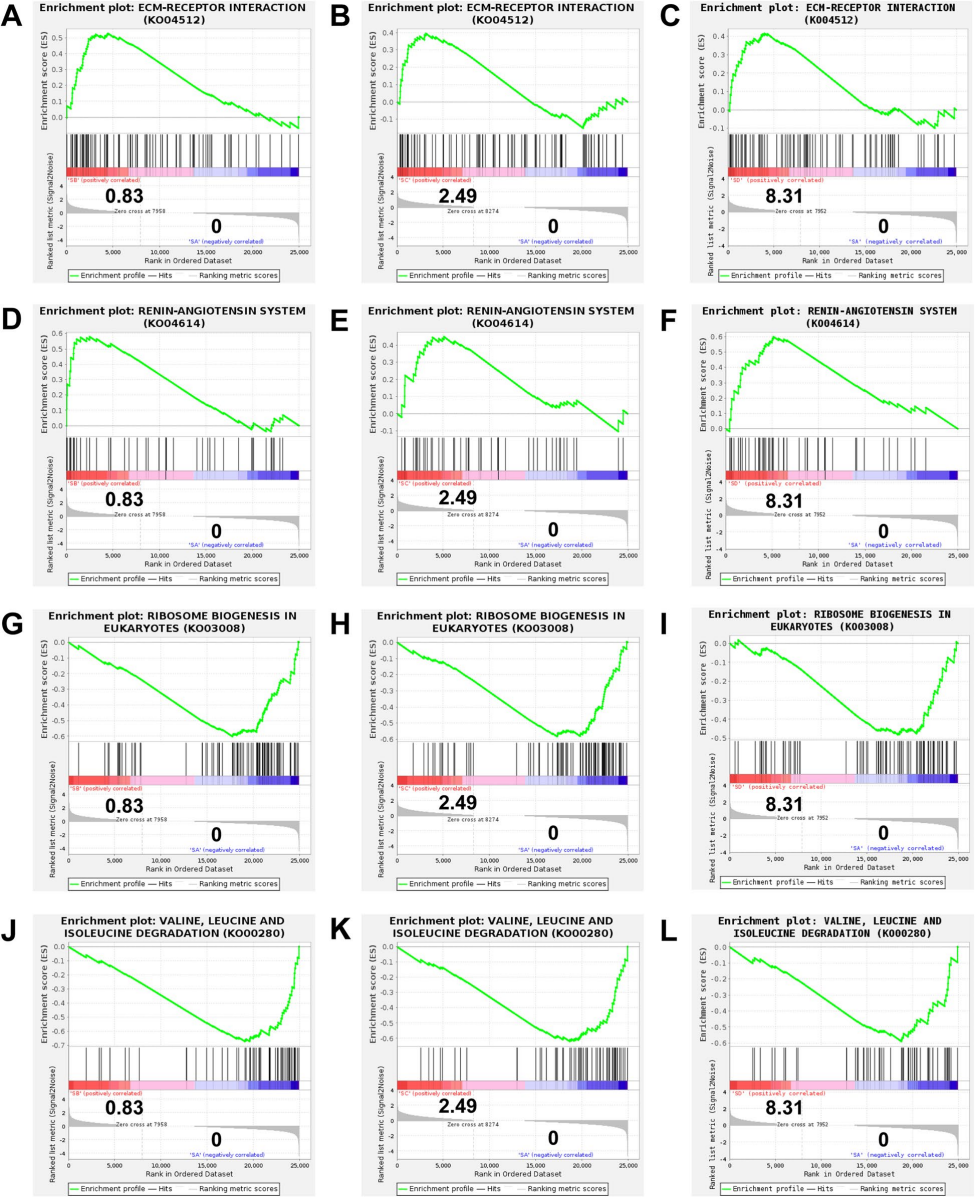
GSEA of nervous system of shrimp under Clothianidin exposure. **A**, 0 vs 0.83 μg/L group, ECM-RECEPTOR_INTERACTION. **B**, 0 vs 2.49 μg/L group, ECM-RECEPTOR_INTERACTION. **C**, 0 vs 8.31 μg/L group, ECM-RECEPTOR_INTERACTION. **D**, 0 vs 0.83 μg/L group, RENIN-ANGIOTENSIN_SYSTEM. **E**, 0 vs 2.49 μg/L group, RENIN-ANGIOTENSIN_SYSTEM. **F**, 0 vs 8.31 μg/L group, RENIN-ANGIOTENSIN_SYSTEM. **G**, 0 vs 0.83 μg/L group, RIBOSOME_BIOGENESIS_IN_EUKARYOTES. **H**, 0 vs 2.49 μg/L group RIBOSOME_BIOGENESIS_IN_EUKARYOTES. **I**, 0 vs 8.31 μg/L group, RIBOSOME_BIOGENESIS_IN_EUKARYOTES. **J**, 0 vs 0.83 μg/L group, VALINE,_LEUCINE_AND_ISOLEUCINE_DEGRADATION. **K**, 0 vs 2.49 μg/L group, VALINE,_LEUCINE_AND_ISOLEUCINE_DEGRADATION. **L**, 0 vs 8.31 μg/L group, VALINE,_LEUCINE_AND_ISOLEUCINE_DEGRADATION

Since GSEA can capture overall and coordinated change patterns, revealing important biological processes related to specific phenotypes or conditions, we further performed GSEA analysis on KEGG-annotated pathways to explore the toxic effects of clothianidin on the nervous system of shrimp. The results showed that in all three exposure groups, the “ECM-RECEPTOR_INTERACTION” and “RENIN-ANGIOTENSIN_SYSTEM” pathways were significantly upregulated, while the “RIBOSOME_BIOGENESIS_IN_EUKARYOTES” and “VALINE, LEUCINE AND ISOLEUCINE DEGRADATION” pathways were significantly downregulated (|NES|> 1 & *P* < 0.05, Fig. 8, A-L).

### PPI

**Fig. 9:**
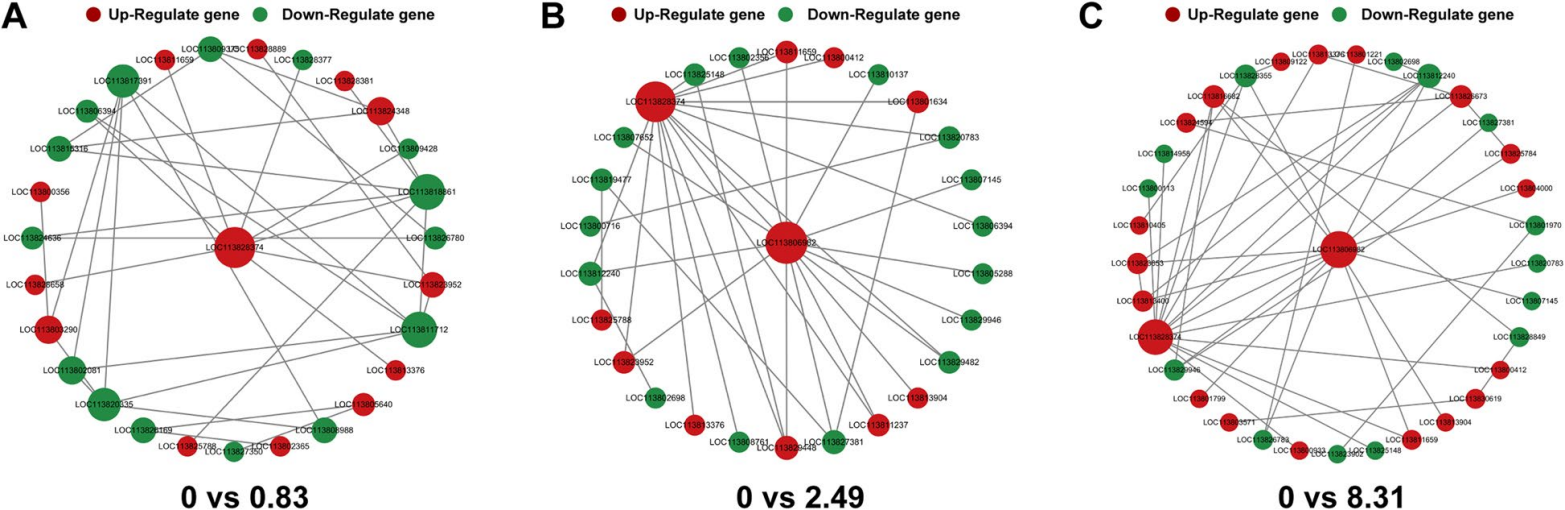
Nodes represent genes, and edges represent the interaction between the two genes (proteins). The size of the node is proportional to the connectivity of the node. The larger the node, the stronger the importance of the gene in the network. The interaction network diagram was constructed using Cytoscape software

All the identified differentially expressed genes (DEGs) were subsequently integrated into the STRING database for protein–protein interaction (PPI) analysis. DEGs with an interaction score higher than 0.4 were selected for further investigation. The comparison between the 0.83 μg/L exposure group and the control group revealed 28 proteins; the comparison between the 2.49 μg/L exposure group and the control group identified 27 proteins; and the comparison between the 8.31 μg/L exposure group and the control group found 34 proteins. As shown in Fig. 9, with increasing clothianidin exposure concentration, upregulated genes gradually dominated the interactions. Specifically, in the 0 vs 0.83 μg/L group, Glyceraldehyde-3-phosphate dehydrogenase (*GAPDH*) and Proliferating cell nuclear antigen (*PCNA*) were the most associated genes, which are involved in glycolysis/gluconeogenesis and DNA replication pathways. In the 0 vs 2.49 μg/L group, Delta-1-pyrroline-5-carboxylate synthase (*P5CS*) and Glyceraldehyde-3-phosphate dehydrogenase (*GAPDH*) were the most associated genes, which belong to the arginine and proline metabolism and glycolysis/gluconeogenesis pathways. In the 0 vs 8.31 μg/L group, Glyceraldehyde-3-phosphate dehydrogenase (*GAPDH*) and Delta-1-pyrroline-5-carboxylate synthase (*P5CS*) were again the most associated genes, involved in glycolysis/gluconeogenesis and arginine and proline metabolism pathways (Table 1).

**Table 1:** The annotation of genes with the highest connectivity in the protein interaction network of each group

Group	Node Name	Node degree	NR description (KO name)	KEGG pathway	Pathway Definition
0vs0.83	*LOC113828374*	9	glyceraldehyde 3-phosphate dehydrogenase (*GAPDH)*	pvm00010	Glycolysis/Gluconeogenesis
	*LOC113811712*	7	proliferating cell nuclear antigen (*PCNA*)	pvm03030	DNA replication
0vs2.49	*LOC113806982*	15	delta-1-pyrroline-5-carboxylate synthetase ((*P5CS*)	pvm00330	Arginine and proline metabolism
	*LOC113828374*	14	glyceraldehyde 3-phosphate dehydrogenase (*GAPDH)*	pvm00010	Glycolysis/Gluconeogenesis
0vs8.31	*LOC113806982*	14	delta-1-pyrroline-5-carboxylate synthetase	pvm00330	Arginine and proline metabolism
	*LOC113828374*	13	glyceraldehyde 3-phosphate dehydrogenase ((*P5CS*)	pvm00010	Glycolysis/Gluconeogenesis

## Discussion

Clothianidin markedly impairs growth performance in *P. vannamei.* After 28 days of exposure, both survival and weight-gain rates declined significantly across all treatment levels, demonstrating that even the lowest concentration tested can suppress growth in aquatic crustaceans. At the highest concentration (8.31 µg/L), shrimp displayed the poorest weight gain yet an elevated condition factor, suggesting stress-driven shifts in body composition. Biochemically, increased lactate dehydrogenase activity pointed to enhanced anaerobic metabolism, while heightened Ca^2^⁺-ATPase and Mg^2^⁺-ATPase activities indicated disturbances in ion homeostasis and water balance (Khan et al. 2020; Duggan 1977). Coupled with suppressed antioxidant and detoxification enzymes, these metabolic disruptions likely altered tissue composition and inflated the condition factor. Collectively, the data highlight a clear, dose-dependent toxic risk of clothianidin to shrimp growth and development.

Clothianidin disrupts antioxidant defense and detoxification capacity in shrimp. The relevant indicators of antioxidant capacity and detoxification metabolism reflect the physiological mechanisms by which shrimp respond to external toxic threats. Hemocyanin—the primary oxygen-transport protein in crustaceans—also serves as a proxy for metabolic activity and immune status because its circulating level reflects overall health (Terwilliger 2015). In our study, clothianidin exposure did not alter hemocyanin concentrations, implying little impact on oxygen transport. This contrasts with reports of imidacloprid-induced hemocyanin suppression in shrimp, suggesting that the two neonicotinoids operate through different toxicological pathways (Fu et al. 2022). RBs are considered an indicator of an organism's stress response, reflecting the respiratory metabolic activity of shrimp when exposed to toxins (Borregaard 1988). Our findings revealed that the respiratory bursts in shrimp exposed to clothianidin were significantly lower than those in the control group, suggesting that clothianidin might impair shrimp respiratory metabolic function. This is consistent with the results observed in shrimp exposed to imidacloprid (Fu et al. 2022), indicating that neonicotinoid insecticides could similarly alter metabolic activities in aquatic organisms, leading to changes in stress responses. SOD is a vital antioxidant enzyme that scavenges ROS to protect cells from oxidative damage (Fridovich 1986). GSH is another essential antioxidant involved in maintaining intracellular redox balance (Battin and Brumaghim 2009). In this experiment, shrimp exposed to clothianidin showed significantly lower SOD and GSH levels compared to the control group, particularly in the high-concentration exposure group, further suggesting that clothianidin induces oxidative stress in shrimp. This finding aligns with the observation that dinotefuran causes a reduction in shrimp GSH levels (Fu et al. 2024). Additionally, GST, an important detoxifying enzyme, plays a crucial role in the metabolism and elimination of toxins (Kumar and Trivedi 2018). In this experiment, the GST activity in shrimp exposed to clothianidin was significantly lower than that of the control group, indicating that clothianidin may impair the detoxification capacity of shrimp. Similarly, exposure to imidacloprid, thiamethoxam, and dinotefuran also reduced GST activity in aquatic organisms, thereby enhancing their toxicity (Fu, et al., 2022a, 2024, 2022b). These results suggest that, like other neonicotinoid insecticides, clothianidin significantly affects the antioxidant and detoxification systems of shrimp, particularly under high-concentration exposure, by inhibiting antioxidant enzyme activity and detoxification capacity, which exacerbates oxidative stress.

Energy metabolism and ion transport regulation are altered as physiological adaptations to clothianidin exposure. Energy metabolism and signal regulation are key mechanisms by which organisms respond to environmental stress. LDH is an important enzyme that reflects the anaerobic glycolysis level during energy metabolism, primarily involved in lactate production (Khan et al. 2020). In this study, the LDH activity in the groups exposed to 0.83 μg/L and 8.31 μg/L of clothianidin was significantly higher than that of the control group, indicating that the shrimp may enhance energy supply in response to clothianidin. This is consistent with the effects of cycloxaprid on energy metabolism in shrimp (Luo et al. 2024). Ca^2^⁺-ATPase and Mg^2^⁺-ATPase regulate the transport of calcium and magnesium ions within cells, involved in various physiological processes, such as signal transduction and muscle contraction (Duggan 1977). In this experiment, the activity of Ca^2^⁺-ATPase and Mg^2^⁺-ATPase was significantly increased in the 8.31 μg/L exposure group, suggesting that the shrimp may regulate cellular signal transduction and stress response by enhancing the transport of calcium and magnesium ions. PKA and PKB are key molecules in signal transduction pathways, involved in regulating cell growth, survival, and metabolic reactions (Song et al. 2005; Taylor et al. 2004). In this study, the activities of PKA and PKB in the clothianidin exposure groups did not show significant changes, indicating that clothianidin has a relatively minor effect on these two protein kinases, potentially reflecting that clothianidin primarily regulates shrimp stress responses and metabolism via other pathways. These results suggest that clothianidin exposure may influence the physiological state of shrimp by modulating energy metabolism and signal transduction pathways. It is possible that shrimp initiate adaptive responses to cope with clothianidin-induced stress by enhancing energy metabolic activity and regulating intracellular ion transport. Such physiological adjustments may reflect a general strategy to maintain homeostasis under toxic conditions.

Clothianidin induces neurotoxicity by disrupting neurotransmitter levels and cholinergic signaling. Neonicotinoid insecticides, such as imidacloprid, mimic ACh by binding to acetylcholine receptors, leading to continuous excitation of pests, which ultimately causes paralysis and death (Anadón et al. 2020). In this experiment, clothianidin exposure significantly decreased acetylcholine levels in shrimp, especially in the 0.83 μg/L and 8.31 μg/L exposure groups, where acetylcholine concentrations were significantly lower than in the control group. This is consistent with the known mode of action of neonicotinoid insecticides. The observed reduction in acetylcholine levels suggests that clothianidin disrupts cholinergic signaling in *P. vannamei,* potentially leading to neurophysiological dysfunctions. These findings provide further evidence of clothianidin-induced neurotoxicity in crustaceans. Meanwhile, AChE activity also showed significant changes in the clothianidin treatment groups at different concentrations. Clothianidin exposure resulted in a marked change in acetylcholinesterase activity, which may further confirm its mechanism through acetylcholine receptor interaction. As the enzyme responsible for breaking down acetylcholine, the activity of acetylcholinesterase is closely related to changes in acetylcholine levels (Quinn 1987), suggesting that clothianidin may inhibit the breakdown of acetylcholine, thereby enhancing its action and affecting the shrimp's nervous system function. Furthermore, DA, another important neurotransmitter (Klein et al. 2019), also showed significant changes in the exposure groups.

High-dose clothianidin disrupts physiological homeostasis in *P. vannamei*, and high-throughput transcriptomic profiling clearly captures the cascade of concentration-dependent changes in gene expression, signaling pathways, and regulatory networks that underlie this stress response (Lowe et al. 2017). We observed a counter-intuitive pattern: as clothianidin concentrations rose, the total number of differentially expressed genes (DEGs) declined. Such a reduction is often interpreted as “transcriptional silencing,” a phenomenon reported in crustaceans and insects under severe toxic stress. When exposure exceeds the organism's compensatory threshold, broad repression of transcriptional activity—via energy depletion, oxidative damage to transcriptional machinery, or stress-induced shutdown of translation initiation—can occur. Consequently, only a core set of stress-tolerance and survival genes remains active, while many non-essential pathways are downregulated. This shrinkage of the DEG repertoire therefore signals diminished transcriptional plasticity and a loss of adaptive capacity (Corre and Kremer 2012; Sagi et al. 2013; Tomoyasu et al. 2008). At the physiological level, stronger disturbances—such as redox imbalance, ionic dysregulation, and mitochondrial impairment—likely overwhelmed detoxification and antioxidant defenses, further accelerating the collapse of gene-regulatory flexibility. Together, these data indicate that high clothianidin doses push shrimp beyond their homeostatic limits, suppressing global gene expression and compromising long-term survival and growth.

KEGG and GO enrichment analyses demonstrate that clothianidin disrupts key metabolic, developmental, and stress-response pathways, underscoring its significant impact on the physiological state of shrimp. In the KEGG enrichment analysis, several pathways related to metabolism and cellular functions were significantly enriched. The protein digestion and absorption pathway mainly involves the synthesis and degradation of intracellular proteins, which is crucial for maintaining normal metabolism in organisms (Bhutia and Ganapathy 2018). Enrichment of the renin-angiotensin system is associated with water-salt balance and blood pressure regulation in the body (Upadrasta and Madempudi 2016), suggesting that clothianidin may affect the physiological homeostasis of shrimp. The enrichment of phototransduction pathways indicates that clothianidin might influence the behavior and neural functions of shrimp by interfering with light perception and nervous system regulation. More importantly, the enrichment of the glutathione metabolism pathway further validates that clothianidin induces oxidative stress in shrimp cells. In the highest-concentration group (8.31 µg/L), pathway enrichment analysis highlighted key metabolic routes—including glycolysis/gluconeogenesis and dicarboxylate metabolism—that are central to ATP generation and the provision of metabolic intermediates (Cori and Cori 1946). Their disturbance suggests that clothianidin compromises cellular energy production and intermediary metabolism in shrimp. The enrichment of pathways related to the cell cycle and DNA replication suggests potential effects of clothianidin on cell division and gene replication processes (Copani et al. 2007), which may interfere with normal growth and reproduction in shrimp. GO enrichment revealed several terms linked to cellular function and stress response. Notably, terms associated with exoskeletal structural components and structural-molecule activity were over-represented, suggesting that clothianidin may impair exoskeleton formation and integrity, thereby hindering normal growth and development in shrimp (Watanabe et al. 2003). Enrichment of pathways related to cellular responses to toxic substances and oxidative detoxification further supports that clothianidin affects the physiological state of shrimp by increasing oxidative stress and detoxification burden. Pathway enrichment shifted as clothianidin concentration increased. Low-dose exposure chiefly highlighted metabolic routes and cell-signalling cascades, whereas high-dose exposure was dominated by oxidative-stress responses and xenobiotic detoxification. This concentration-dependent shift implies that higher clothianidin levels intensify toxicity, provoking pronounced alterations in metabolism and cellular function in shrimp.

GSEA indicates that clothianidin simultaneously disrupts ribosome function and amino-acid metabolism while implicating neurotoxic pathways in shrimp. The ECM-receptor interaction pathway plays a crucial role in the interaction between cells and the extracellular matrix, regulating cell adhesion, migration, and signal transduction, which affects tissue structure and homeostasis (Nersisyan et al. 2021). The renin-angiotensin system is a key pathway in fluid balance regulation, involving the control of blood pressure, cell proliferation, and apoptosis (Paul et al. 2006). In this study, these pathways were consistently up-regulated across all exposure groups, indicating that clothianidin may enhance intercellular communication and signal transduction by facilitating extracellular signal transmission and reinforcing the extracellular matrix. Additionally, the ribosome biogenesis in eukaryotes and valine, leucine, and isoleucine degradation pathways were significantly downregulated in all exposure groups. Ribosomes are crucial for protein synthesis, and inhibition of ribosome biogenesis would impact protein production and cellular function (Spirin 2019), indicating that clothianidin may weaken the growth and metabolic capacity of shrimp by suppressing intracellular protein synthesis. The downregulation of amino acid metabolism-related pathways also suggests that clothianidin may alter amino acid metabolic processes, affecting cellular energy supply and repair mechanisms, thereby impacting the physiological balance of shrimp (Ling et al. 2023).

Key genes such as *GAPDH* and *P5CS* are centrally involved in shrimp responses to clothianidin exposure. Glyceraldehyde-3-phosphate dehydrogenase (*GAPDH*) is a critical enzyme involved in glycolysis and gluconeogenesis, catalyzing the conversion of glyceraldehyde-3-phosphate to 1,3-bisphosphoglycerate (Kosova et al. 2017). This reaction plays an important role in energy metabolism, especially when cellular energy demand is high, where*GAPDH* is crucial for providing energy to the cells. Delta-1-pyrroline-5-carboxylate synthetase (*P5CS*) is an enzyme involved in amino acid metabolism, primarily participating in the metabolism of arginine and proline, and is an essential component of the proline biosynthesis pathway (Feng et al. 2022). Proline plays a significant role in stress response, antioxidant defense, and cellular protection (Liang et al. 2013). In the PPI analysis, both*GAPDH* and *P5CS* showed high connectivity in different treatment groups, especially in the 0 vs 0.83 μg/L and 0 vs 2.49 μg/L groups. This suggests that these two genes may play a vital physiological regulatory role under clothianidin exposure through interactions with other metabolism-related genes. In particular, the central role of *GAPDH* in metabolic pathways reflects its core function in cellular energy metabolism, while *P5CS* may enhance the adaptation to environmental stress by regulating amino acid metabolism. These results further suggest that clothianidin may exacerbate shrimp's response to environmental stress by interfering with energy and amino acid metabolic pathways.

Compared with other aquatic organisms such as zebrafish (*Danio rerio*), rainbow trout (*Oncorhynchus mykiss*), and Northern Clearwater Crayfish (*Orconectes propinquus*), *P. vannamei* exhibited distinct physiological and transcriptomic responses to clothianidin exposure (Miles et al. 2017; Dogan et al. 2022; Patil and D'souza 2024). While neonicotinoid exposure in fish often results in developmental toxicity, immune suppression, and hepatotoxicity (Morrissey et al. 2015; Malhotra et al. 2021), our study revealed that shrimp responded with marked oxidative stress, neurotransmitter imbalance, and disruption of neural signaling pathways, particularly at low and moderate concentrations. Previous studies on imidacloprid and thiamethoxam have reported acetylcholinesterase inhibition and behavioral abnormalities in fish (Naiel et al. 2020; Hussain et al. 2022); however, *P. vannamei* demonstrated more consistent and dose-dependent transcriptomic alterations in both neurotransmission and detoxification pathways under clothianidin exposure.

**Fig. 10:**
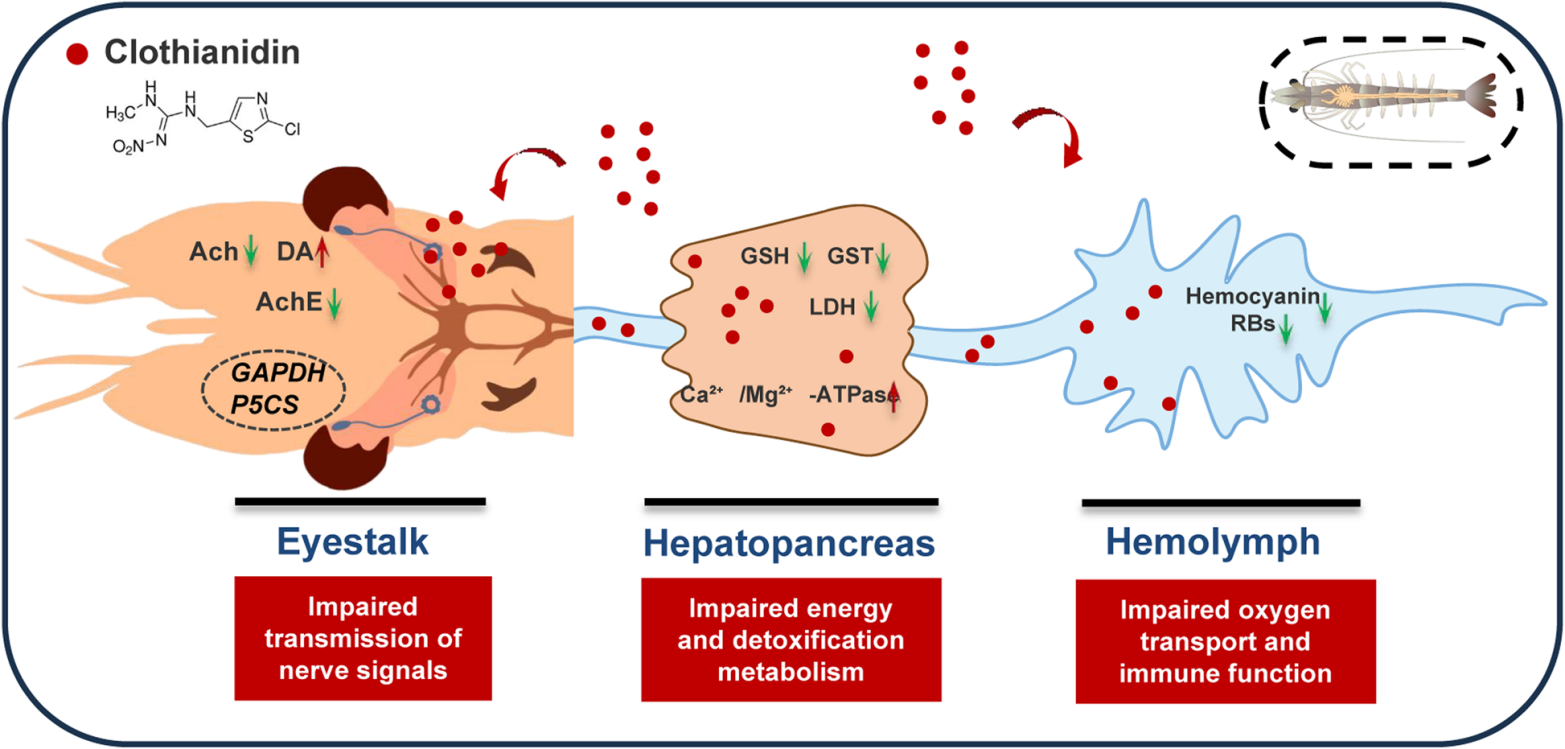
Diagram of the mechanism by which clothianidin affects shrimp (*Penaeus vannamei*)

In summary, this study reveals the multifaceted effects of clothianidin on *P. vannamei* (Fig. 10). Clothianidin exposure significantly inhibited the growth performance of shrimp, increased oxidative stress, and disrupted their antioxidant and detoxification capacities. Meanwhile, clothianidin further impacted shrimp physiology by altering energy metabolism and signal transduction pathways, as well as affecting the balance of neurotransmitters. Among the various responses, changes in ACh and AChE levels emerged as particularly sensitive biomarkers of clothianidin-induced neurotoxicity. Transcriptome analysis revealed that clothianidin regulates multiple biological pathways, significantly altering the gene expression profile of shrimp, providing new insights into the toxicological mechanisms of clothianidin. Future studies could further investigate the long-term effects of clothianidin on the nervous system and reproductive health, and validate the roles of key genes.

## Conclusions

This study represents the first systematic investigation into the potential toxic effects of clothianidin on decapod crustaceans, specifically *P. vannamei.* The results demonstrate that clothianidin exposure significantly impacted the growth performance of shrimp, with a marked decline in survival and weight gain rates, particularly at higher concentrations. Additionally, clothianidin induced oxidative stress and inhibited the activity of antioxidant and detoxification enzymes, thereby impairing the shrimp's physiological defense systems. Transcriptomic analysis revealed that clothianidin affected shrimp physiology by regulating several metabolic and neurotransmission-related pathways. Notably, *GAPDH* and *P5CS* exhibited significant interactions within multiple pathways, suggesting their key roles in the toxic response to clothianidin. Furthermore, the consistent and concentration-dependent changes observed in ACh and AChE levels highlight their potential as sensitive biomarkers for early detection of clothianidin-induced neurotoxicity. Overall, this study provides new insights into the toxicological mechanisms of clothianidin in aquatic organisms and offers crucial data for evaluating its ecological risks and identifying potential biomarkers. However, the specific molecular mechanisms of clothianidin and its long-term effects warrant further investigation.

## Materials and methods

### Pesticides and shrimp

Clothianidin (CLO, CAS 210880–92–5, HPLC purity ≥ 99%) was purchased from Shanghai Yuanye Biological Technology Co., Ltd. (Shanghai, China). Clothianidin was dissolved in DMSO and stored in a brown glass bottle wrapped with aluminum foil to prevent photodegradation, then diluted to the required concentrations. The stock solution was kept at 4 °C and replenished every two days.

The juvenile shrimp (approximately P10) were obtained from Hainan Blue Ocean Biotechnology Co., Ltd. (Wenchang, Hainan Province, China). Prior to exposure, the shrimp were acclimated in the laboratory culture tanks for four weeks. Healthy juveniles (0.32 ± 0.01 g) were then randomly distributed into glass tanks (60 cm × 30 cm × 35 cm). The water salinity was maintained at 28–30‰, pH at 7.0–7.5, and temperature at 27 ± 1 °C, with continuous aeration for 24 h to ensure that dissolved oxygen levels remained above 6 mg/L.

### Experimental design

The 96-h acute toxicity experiment was set with target exposure concentrations of 0, 20, 50, 100, 200, and 400 μg/L of clothianidin. The concentrations refer to the actual content of clothianidin (purity ≥ 99%) used in the exposure solutions. Twenty juvenile shrimp of similar size and shape were randomly placed in each glass tank, with three replicate groups for each concentration. Survival was recorded at 24, 48, 72, and 96 h to determine the median lethal concentration (LC_50_). Every other day, half of the clothianidin solution was replaced with fresh solution of the same concentration, and any dead shrimp were promptly removed.

Based on the 96-h LC_50_ of clothianidin (24.93 μg/L) for juvenile shrimp and the range of concentrations detected globally in aquatic environments (< 0.025—3.5 μg/L), three clothianidin concentrations were selected for the chronic toxicity study: 1/3 (8.31 μg/L), 1/10 (2.49 μg/L), and 1/30 (0.83 μg/L) of the 96-h LC_50_. The concentrations refer to the actual content of clothianidin (purity ≥ 99%) used in the exposure solutions. Additionally, a control group was set up, with four replicates for each concentration. The tank size, water volume, aeration, and temperature conditions used in the chronic experiment were consistent with those used in the acute toxicity test. In this chronic toxicity study, 30 shrimp were used per replicate. During the experiment, half of the water was changed daily, with dead shrimp and residual feed being removed, and every 4 days, the entire water was replaced to maintain consistency with the chronic experiment. Simultaneously, clothianidin solution was added to the water based on the volume of water exchanged, ensuring the experimental concentrations were maintained. Commercial feed was provided four times daily, with a feeding rate of 5% of the shrimp's body weight. No obvious abnormalities in feeding behavior were observed throughout the exposure period; therefore, no specific feeding behavior was recorded.

### Clothianidin concentration analysis

The Sciex/Qtrap6500 + Liquid Chromatography Triple Quadrupole Mass Spectrometry System was provided by Hercules AB SCIEX. The ultrapure water system was produced by QingdaoFule Technology Co., Ltd. The analytical balance was from A&D Company. The XH-C vortex mixer was manufactured by Jintan Baida Xinbao Instrument Factory. The 0.22 μm syringe filter membrane for the aqueous phase was provided by Tianjin Jinteng Laboratory Equipment Co., Ltd. HPLC‒MS grade methanol was purchased from Shanghai Aladdin Biochemical Technology Co., Ltd., and HPLC‒MS grade acetonitrile was also obtained from Shanghai Aladdin Biochemical Technology Co., Ltd.

During the experiment, 500 ml water samples were collected from each experimental group, filtered through 0.22 µm membranes to remove particulate matter, and then transferred to 2 ml glass vials for analysis. The concentration of clothianidin was measured using a hybrid triple quadrupole-linear ion trap mass spectrometer (6500 + QTRAP® LC–MS/MS system, AB Sciex, USA). Chromatographic separation was achieved using a Kinetex® 1.7 µm C18 100 Å liquid chromatography column (150 mm × 2.1 mm, part number: 00 F-4475-AN, serial number: H22-078119, Phenomenex, USA). The mobile phase A was a 0.1% formic acid aqueous solution, and phase B was a 0.1% formic acid acetonitrile solution. The flow rate was set to 0.3 ml/min, with a column temperature of 40 °C and an auto-sampler temperature of 4 °C. The gradient elution program was as follows: from 0 to 3 min, the B-phase proportion increased from 5 to 90%; from 3 to 5 min, 90% B-phase was maintained; from 5 to 5.1 min, the B-phase proportion decreased from 90 to 5%; from 5.1 to 6 min, 5% B-phase was maintained. Data were collected using positive ion electrospray ionization (ESI +) mode in multiple reaction monitoring (MRM) mode, with a parent ion m/z of 250 and product ions m/z of 132 and 169.1. The measured exposure concentrations were 1.10 ± 0.01 μg/L, 3.76 ± 0.15 μg/L, and 9.25 ± 0.27 μg/L (*n* = 3).

### Sample collection

After 28 days of exposure, the shrimp were fasted for 24 h, then all the shrimp were removed from the glass aquaculture tank. After anesthesia with an ice bath, the shrimp were blotted dry using sterile absorbent paper, and body weight (measured with an electronic balance with an accuracy of 0.01 g) and body length (measured with a caliper with an accuracy of 0.1 mm) were precisely determined. A sterile 1 mL plastic syringe (26G needle) was used to collect 200 μL of mixed solution from the heart sinus by direct puncture. The syringe was preloaded with 200 μL of pre-cooled (4 °C) citrate anticoagulant (sodium citrate·2H₂O 30.8 mmol/L, pH 7.4), and 200 μL of mixed solution was accurately collected from each individual. The hepatopancreas and eyestalk tissues were dissected and transferred to 1.5 mL enzyme-free centrifuge tubes, which were snap-frozen in liquid nitrogen for 2 h and then stored at −80 °C for long-term preservation. The growth performance parameters were calculated as follows: Survival rate (SR, %) = (final number of surviving individuals/initial number of individuals) × 100; Weight gain rate (WGR, %) = (final body weight—initial body weight)/initial body weight × 100; Condition factor (CF, g/cm^3^) = body weight/body length^3^ × 100; Hepatosomatic index (HSI, %) = hepatopancreas wet weight/body weight × 100.

### Biochemical analysis

To assess the toxicological effects of clothianidin exposure on juvenile *P. vannamei* this study systematically analyzed the dynamic changes in biomarker levels in hemolymph and hepatopancreas. Hemolymph samples were pre-cooled at 4 °C and centrifuged at 4000 rpm for 10 min using a SIGMA centrifuge (Germany). Hemocyanin concentration was determined by ultraviolet spectrophotometry: 30 μL of the supernatant was transferred to a 96-well plate and 270 μL of ultrapure water was added and mixed gently. The absorbance at 335 nm was measured and the hemocyanin concentration was calculated using the formula E 335 nm (mg/mL) = 2.83 × OD 335 nm where E represents the hemocyanin concentration and 2.83 is the characteristic extinction coefficient for hemocyanin following the method established byNickerson and Van Holde in 1971 (Nickerson and Holde 1971). Respiratory burst activity was measured by the nitro blue tetrazolium (NBT) formaldehyde reduction method to quantify superoxide anion generation, with analysis conducted at a wavelength of 630 nm (methodology based on Song and Hsieh, ).

Hepatopancreas samples were collected using a mixed sampling strategy, with 4 replicates per group (*n* = 4), each containing tissue samples from 3 individuals. The tissue samples were homogenized in pre-chilled physiological saline (0.86% NaCl) at a weight-to-volume ratio of 1:9. After preparing a 10% tissue homogenate, the samples were centrifuged at 3000 rpm for 10 min at 4 °C, and the supernatants were collected for biochemical analysis. The following assays were conducted using commercial kits from the Nanjing Jiancheng Bioengineering Institute: superoxide dismutase (SOD, A001-3–2, WST-1 method), glutamine synthetase (GS, A047-1–1, colorimetric method), reduced glutathione (GSH, A006-2–1, microplate method), glutathione S-transferase (GST, A004-1–1, colorimetric method), lactate dehydrogenase (LDH, A020-2–2, microplate method), ATPase (Ca^2+^Mg^2+^-ATPase, A016-2–1), protein kinase A (PKA, H233-1–1), protein kinase B (PKB, H233-3), acetylcholine (ACh, A105-1–1, microplate method), acetylcholinesterase (AChE, H529-1–1), and dopamine (DA, H170-1–1). 5-Hydroxytryptamine (5-HT) activity was determined using an ELISA kit from Shanghai Fantai Biotechnology Co., Ltd., following the manufacturer's instructions.

### Transcriptome sequencing and analysis

To further elucidate the toxicological mechanisms of clothianidin exposure on *P. vannamei*, this study selected eye stalk tissue samples from the control group and three exposed groups (three biological replicates per group, for a total of 12 samples) for transcriptomic sequencing analysis. The experimental procedure was as follows: Total RNA from the tissues was extracted using the TRNzol Universal reagent (Invitrogen, USA) according to the manufacturer's instructions. RNA purity was assessed using a Nanodrop 2000 spectrophotometer, and RNA integrity was verified by 1% agarose gel electrophoresis. The RNA integrity number (RIN) was determined using the Agilent 2100 Bioanalyzer system. For samples that met the library construction requirements (total RNA ≥ 1 μg, concentration ≥ 35 ng/μL), library preparation was performed using the Illumina NovaSeq 6000 sequencing platform. Specifically, sequencing libraries were prepared using the TruSeq PE Cluster Kit v3-cBotHS kit (Illumina, USA) following the standard protocol, and paired-end (PE150) raw sequencing data was generated on the Illumina NovaSeq platform. The raw fastq format data was preprocessed using Perl scripts, with low-quality reads containing adapter sequences removed to obtain high-quality clean reads. All sequences have been submitted to the NCBI SRA (PRJNA1233436).

The clean reads were then aligned to the *P. vannamei* reference genome (GCF_024377395.1) using HISAT2 (v2.0.5), and gene expression quantification was performed with RSEM software (v1.3.3). Differentially expressed genes (DEGs) were selected using the criteria of *p*-value < 0.05 and |log_2_(FC)|> 2.

To further analyze the functional roles of the differentially expressed genes, this study utilized Gene Ontology (GO) functional annotation and Kyoto Encyclopedia of Genes and Genomes (KEGG) pathway enrichment analysis to identify significantly enriched biological processes and metabolic pathways. Gene Set Enrichment Analysis (GSEA, v4.1.0) was performed on all genes to identify enriched pathways, and the results were visualized with pathway enrichment plots. Additionally, a protein–protein interaction (PPI) network of the DEGs was constructed based on the STRING database (v12.0), retaining interactions with a combined score ≥ 0.4. Finally, the network topology was visualized using Cytoscape software (v3.10.1) to identify key regulatory genes and their interaction networks under clothianidin exposure.

### Statistical analysis

All data were analyzed using one-way analysis of variance (ANOVA), followed by Duncan's multiple comparison test using GraphPad Prism software (GraphPad Software, La Jolla, CA). Data are expressed as the mean ± standard error (SE). *P* < 0.05 was regarded as statistically significant.

## Supplementary Information

Supplementary Material 1.

## Data Availability

Not applicable.

## Abbreviations

Ach: Acetylcholine
AChE: Acetylcholinesterase
CF: Condition factor
CLO: Clothianidin
DA: Dopamine
DEGs: Differentially expressed genes
*GAPDH*: Glyceraldehyde-3-phosphate dehydrogenase
GO: Gene Ontology
GS: Glutamine synthetase
GSEA: Gene Set Enrichment Analysis
GSH: Glutathione
GST: Glutathione S-transferase
HIS: Hepatosomatic index
KEGG: Kyoto Encyclopedia of Genes and Genomes
LC_50_: Median lethal concentration
LDH: Lactate dehydrogenase
nAChRs: Nicotinic acetylcholine receptors
NBT: Nitro blue tetrazolium
*P. vannamei*: *Penaeus vannamei*
*P5CS*: Delta-1-pyrroline-5-carboxylate synthetase
PKA: Protein kinase A
PKB: Protein kinase B
PPI: Protein-protein interaction
RBs: Respiratory bursts
RIN: RNA integrity number
SOD: Superoxide dismutase
SR: Survival rate
WGR: Weight gain rate
5-HT: 5-Hydroxytryptamine
