# Supplementary material for: Impact of clothianidin exposure on the growth, metabolism, and neurological function of Penaeus vannamei
Source: Stress Biol. 2025 Nov 2;5(1):67. doi: 10.1007/s44154-025-00259-0 (PMC12579615; doi:10.1007/s44154-025-00259-0)
Supplement: Supplementary file 1 — Supplementary Material 1. [file 44154_2025_259_MOESM1_ESM.docx]

**Supplementary data for** **“Impact of Clothianidin Exposure on the Growth, Metabolism, and Neurological Function of *Penaeus vannamei*”**

Zhi Luo ^a#^, Zhen-Fei Li ^b#^, Zhi-Yu Lin ^b^, Zhen-Qiang Fu ^c^, Feng-Lu Han ^b^, Er-Chao Li ^a,^ *

^a^ School of Life Sciences, East China Normal University, 500 Dongchuan Road, Shanghai 200241, China

^b^ School of Marine Biology and Fisheries, Hainan University, Haikou, Hainan 570228, China

^c^ School of Marine Science, Sun Yat-sen University, Zhuhai, Guangdong 519082, China

^#^ These authors contributed equally to this work.

* Corresponding authors:

ecli@bio.ecnu.edu.cn (ECL).

**Supplementary Table**

**Table S1**

Significantly enriched KEGG terms in 0.83 vs 0, 2.49 vs 0 and 8.31 vs 0 groups (Corrected *p-*value < 0.05).

| Pathway_ID | Term | Level1 | Level2 | Up | Down | Pvalue |
| --- | --- | --- | --- | --- | --- | --- |
| ***0.83 vs 0 Group*** |  |  |  |  |  |  |
| ko04745 | Phototransduction - fly | Organismal Systems | Sensory system | 9 | 12 | 3.76E-11 |
| ko04611 | Platelet activation | Organismal Systems | Immune system | 8 | 11 | 6.07E-07 |
| ko04974 | Protein digestion and absorption | Organismal Systems | Digestive system | 12 | 2 | 4.56E-06 |
| ko04614 | Renin-angiotensin system | Organismal Systems | Endocrine system | 10 | 0 | 4.57E-05 |
| ko04971 | Gastric acid secretion | Organismal Systems | Digestive system | 1 | 11 | 1.28E-04 |
| ko04978 | Mineral absorption | Organismal Systems | Digestive system | 2 | 8 | 1.81E-04 |
| ko04510 | Focal adhesion | Cellular Processes | Cellular community - eukaryotes | 9 | 11 | 1.89E-04 |
| ko00053 | Ascorbate and aldarate metabolism | Metabolism | Carbohydrate metabolism | 2 | 6 | 2.26E-04 |
| ko04520 | Adherens junction | Cellular Processes | Cellular community - eukaryotes | 0 | 14 | 2.69E-04 |
| ko04640 | Hematopoietic cell lineage | Organismal Systems | Immune system | 8 | 0 | 2.99E-04 |
| ko00480 | Glutathione metabolism | Metabolism | Metabolism of other amino acids | 10 | 2 | 3.44E-04 |
| ko04976 | Bile secretion | Organismal Systems | Digestive system | 2 | 7 | 3.61E-04 |
| ko00140 | Steroid hormone biosynthesis | Metabolism | Lipid metabolism | 1 | 6 | 4.33E-04 |
| ko04512 | ECM-receptor interaction | Environmental Information Processing | Signaling molecules and interaction | 10 | 0 | 4.64E-04 |
| ko00982 | Drug metabolism - cytochrome P450 | Metabolism | Xenobiotics biodegradation and metabolism | 2 | 6 | 5.03E-04 |
| ko04926 | Relaxin signaling pathway | Organismal Systems | Endocrine system | 9 | 1 | 5.61E-04 |
| ko00040 | Pentose and glucuronate interconversions | Metabolism | Carbohydrate metabolism | 1 | 7 | 5.68E-04 |
| ko00980 | Metabolism of xenobiotics by cytochrome P450 | Metabolism | Xenobiotics biodegradation and metabolism | 2 | 6 | 9.03E-04 |
| ko04670 | Leukocyte transendothelial migration | Organismal Systems | Immune system | 0 | 11 | 9.34E-04 |
| ko04919 | Thyroid hormone signaling pathway | Organismal Systems | Endocrine system | 1 | 14 | 1.63E-03 |
| ***2.49 vs 0 Group*** | |  |  |  |  |  |
| ko04213 | Longevity regulating pathway - multiple species | Organismal Systems | Aging | 1 | 25 | 3.98E-15 |
| ko04745 | Phototransduction - fly | Organismal Systems | Sensory system | 16 | 3 | 6.27E-10 |
| ko04141 | Protein processing in endoplasmic reticulum | Genetic Information Processing | Folding, sorting and degradation | 1 | 25 | 2.66E-09 |
| ko04974 | Protein digestion and absorption | Organismal Systems | Digestive system | 9 | 2 | 2.45E-04 |
| ko04622 | RIG-I-like receptor signaling pathway | Organismal Systems | Immune system | 0 | 6 | 6.70E-04 |
| ko00480 | Glutathione metabolism | Metabolism | Metabolism of other amino acids | 7 | 4 | 7.17E-04 |
| ko04512 | ECM-receptor interaction | Environmental Information Processing | Signaling molecules and interaction | 8 | 1 | 1.18E-03 |
| ko04668 | TNF signaling pathway | Environmental Information Processing | Signal transduction | 1 | 6 | 7.06E-03 |
| ko04918 | Thyroid hormone synthesis | Organismal Systems | Endocrine system | 3 | 3 | 1.26E-02 |
| ko04614 | Renin-angiotensin system | Organismal Systems | Endocrine system | 5 | 1 | 1.46E-02 |
| ko00730 | Thiamine metabolism | Metabolism | Metabolism of cofactors and vitamins | 2 | 1 | 1.50E-02 |
| ko04926 | Relaxin signaling pathway | Organismal Systems | Endocrine system | 7 | 0 | 1.79E-02 |
| ko04611 | Platelet activation | Organismal Systems | Immune system | 9 | 1 | 2.07E-02 |
| ko00790 | Folate biosynthesis | Metabolism | Metabolism of cofactors and vitamins | 3 | 2 | 2.28E-02 |
| ko04610 | Complement and coagulation cascades | Organismal Systems | Immune system | 3 | 1 | 2.30E-02 |
| ko00590 | Arachidonic acid metabolism | Metabolism | Lipid metabolism | 1 | 4 | 2.64E-02 |
| ko04975 | Fat digestion and absorption | Organismal Systems | Digestive system | 2 | 2 | 3.02E-02 |
| ko04972 | Pancreatic secretion | Organismal Systems | Digestive system | 4 | 5 | 3.14E-02 |
| ko04151 | PI3K-Akt signaling pathway | Environmental Information Processing | Signal transduction | 8 | 4 | 3.84E-02 |
| ko04929 | GnRH secretion | Organismal Systems | Endocrine system | 3 | 0 | 7.20E-02 |
| ***8.31 vs 0 Group*** | |  |  |  |  |  |
| ko04745 | Phototransduction - fly | Organismal Systems | Sensory system | 16 | 5 | 7.86E-13 |
| ko04213 | Longevity regulating pathway - multiple species | Organismal Systems | Aging | 0 | 15 | 1.27E-06 |
| ko00232 | Caffeine metabolism | Metabolism | Biosynthesis of other secondary metabolites | 2 | 2 | 4.52E-04 |
| ko04141 | Protein processing in endoplasmic reticulum | Genetic Information Processing | Folding, sorting and degradation | 0 | 15 | 9.35E-04 |
| ko00983 | Drug metabolism - other enzymes | Metabolism | Xenobiotics biodegradation and metabolism | 5 | 4 | 1.34E-03 |
| ko04622 | RIG-I-like receptor signaling pathway | Organismal Systems | Immune system | 0 | 5 | 2.51E-03 |
| ko00790 | Folate biosynthesis | Metabolism | Metabolism of cofactors and vitamins | 5 | 1 | 2.88E-03 |
| ko04974 | Protein digestion and absorption | Organismal Systems | Digestive system | 6 | 2 | 5.79E-03 |
| ko04512 | ECM-receptor interaction | Environmental Information Processing | Signaling molecules and interaction | 6 | 1 | 8.20E-03 |
| ko00730 | Thiamine metabolism | Metabolism | Metabolism of cofactors and vitamins | 3 | 0 | 1.06E-02 |
| ko00053 | Ascorbate and aldarate metabolism | Metabolism | Carbohydrate metabolism | 3 | 2 | 1.18E-02 |
| ko00860 | Porphyrin metabolism | Metabolism | Metabolism of cofactors and vitamins | 2 | 3 | 1.27E-02 |
| ko00670 | One carbon pool by folate | Metabolism | Metabolism of cofactors and vitamins | 2 | 1 | 1.45E-02 |
| ko00980 | Metabolism of xenobiotics by cytochrome P450 | Metabolism | Xenobiotics biodegradation and metabolism | 4 | 1 | 2.59E-02 |
| ko04977 | Vitamin digestion and absorption | Organismal Systems | Digestive system | 2 | 4 | 2.80E-02 |
| ko04614 | Renin-angiotensin system | Organismal Systems | Endocrine system | 5 | 0 | 3.11E-02 |
| ko00480 | Glutathione metabolism | Metabolism | Metabolism of other amino acids | 7 | 0 | 3.47E-02 |
| ko00591 | Linoleic acid metabolism | Metabolism | Lipid metabolism | 3 | 0 | 4.09E-02 |
| ko04668 | TNF signaling pathway | Environmental Information Processing | Signal transduction | 0 | 5 | 4.80E-02 |
| ko04640 | Hematopoietic cell lineage | Organismal Systems | Immune system | 4 | 0 | 5.45E-02 |

**Table S2**

Significantly enriched GO terms in 0.83 vs 0, 2.49 vs 0 and 8.31 vs 0 groups (Corrected *p-*value < 0.05).

| GO_ID | Category | Term | Up | Down | Pvalue |
| --- | --- | --- | --- | --- | --- |
| ***0.83 vs 0 Group*** | | |  |  |  |
| GO:0042302 | MF | structural constituent of cuticle | 74 | 8 | 6.24E-49 |
| GO:0005198 | MF | structural molecule activity | 75 | 8 | 1.12E-32 |
| GO:0042555 | CC | MCM complex | 0 | 3 | 3.16E-04 |
| GO:0006270 | BP | DNA replication initiation | 0 | 3 | 8.43E-04 |
| GO:0098869 | BP | cellular oxidant detoxification | 2 | 3 | 8.73E-04 |
| GO:0097237 | BP | cellular response to toxic substance | 2 | 3 | 1.18E-03 |
| GO:1990748 | BP | cellular detoxification | 2 | 3 | 1.18E-03 |
| GO:0006979 | BP | response to oxidative stress | 2 | 3 | 1.37E-03 |
| GO:0098754 | BP | detoxification | 2 | 3 | 1.37E-03 |
| GO:0030855 | BP | epithelial cell differentiation | 2 | 1 | 1.74E-03 |
| GO:0016810 | MF | hydrolase activity, acting on carbon-nitrogen (but not peptide) bonds | 3 | 4 | 1.88E-03 |
| GO:0006048 | BP | UDP-N-acetylglucosamine biosynthetic process | 2 | 0 | 1.93E-03 |
| GO:0006189 | BP | 'de novo' IMP biosynthetic process | 0 | 2 | 1.93E-03 |
| GO:0015767 | BP | lactose transport | 0 | 2 | 1.93E-03 |
| GO:0015768 | BP | maltose transport | 0 | 2 | 1.93E-03 |
| GO:0015770 | BP | sucrose transport | 0 | 2 | 1.93E-03 |
| GO:0015771 | BP | trehalose transport | 0 | 2 | 1.93E-03 |
| GO:0004610 | MF | phosphoacetylglucosamine mutase activity | 2 | 0 | 2.17E-03 |
| GO:0006040 | BP | amino sugar metabolic process | 6 | 0 | 2.38E-03 |
| GO:0070887 | BP | cellular response to chemical stimulus | 5 | 3 | 2.61E-03 |
| ***2.49 vs 0 Group*** | | |  |  |  |
| GO:0042302 | MF | structural constituent of cuticle | 87 | 8 | 3.06E-63 |
| GO:0005198 | MF | structural molecule activity | 88 | 8 | 2.47E-43 |
| GO:0005576 | CC | extracellular region | 11 | 20 | 1.69E-10 |
| GO:0010466 | BP | negative regulation of peptidase activity | 1 | 10 | 8.35E-09 |
| GO:0052547 | BP | regulation of peptidase activity | 1 | 10 | 1.12E-08 |
| GO:0045861 | BP | negative regulation of proteolysis | 1 | 10 | 1.98E-08 |
| GO:0051346 | BP | negative regulation of hydrolase activity | 1 | 10 | 1.98E-08 |
| GO:0043086 | BP | negative regulation of catalytic activity | 1 | 10 | 8.97E-08 |
| GO:0044092 | BP | negative regulation of molecular function | 1 | 10 | 1.13E-07 |
| GO:0030162 | BP | regulation of proteolysis | 1 | 10 | 1.75E-07 |
| GO:0032269 | BP | negative regulation of cellular protein metabolic process | 1 | 10 | 2.67E-07 |
| GO:0051248 | BP | negative regulation of protein metabolic process | 1 | 10 | 3.27E-07 |
| GO:0030414 | MF | peptidase inhibitor activity | 1 | 10 | 1.22E-06 |
| GO:0061134 | MF | peptidase regulator activity | 1 | 10 | 1.22E-06 |
| GO:0051336 | BP | regulation of hydrolase activity | 2 | 10 | 1.69E-06 |
| GO:0006508 | BP | proteolysis | 8 | 15 | 3.56E-06 |
| GO:0004857 | MF | enzyme inhibitor activity | 1 | 10 | 7.18E-06 |
| GO:0048523 | BP | negative regulation of cellular process | 3 | 13 | 1.76E-05 |
| GO:0051172 | BP | negative regulation of nitrogen compound metabolic process | 1 | 10 | 1.05E-04 |
| GO:0050790 | BP | regulation of catalytic activity | 2 | 10 | 1.06E-04 |
| ***8.31 vs 0 Group*** | | |  |  |  |
| GO:0042302 | MF | structural constituent of cuticle | 91 | 6 | 4.72E-68 |
| GO:0005198 | MF | structural molecule activity | 94 | 6 | 1.93E-49 |
| GO:0007602 | BP | phototransduction | 2 | 7 | 6.96E-07 |
| GO:0009583 | BP | detection of light stimulus | 2 | 7 | 6.96E-07 |
| GO:0009581 | BP | detection of external stimulus | 2 | 7 | 8.52E-07 |
| GO:0009582 | BP | detection of abiotic stimulus | 2 | 7 | 8.52E-07 |
| GO:0051606 | BP | detection of stimulus | 2 | 7 | 1.04E-06 |
| GO:0009605 | BP | response to external stimulus | 4 | 7 | 5.81E-06 |
| GO:0071482 | BP | cellular response to light stimulus | 0 | 4 | 1.07E-05 |
| GO:0008020 | MF | G protein-coupled photoreceptor activity | 0 | 4 | 2.05E-05 |
| GO:0007186 | BP | G protein-coupled receptor signaling pathway | 5 | 8 | 2.14E-05 |
| GO:0009416 | BP | response to light stimulus | 2 | 7 | 2.34E-05 |
| GO:0009314 | BP | response to radiation | 2 | 7 | 4.77E-05 |
| GO:0071478 | BP | cellular response to radiation | 0 | 4 | 6.08E-05 |
| GO:0009881 | MF | photoreceptor activity | 2 | 7 | 1.07E-04 |
| GO:0071214 | BP | cellular response to abiotic stimulus | 0 | 4 | 1.38E-04 |
| GO:0104004 | BP | cellular response to environmental stimulus | 0 | 4 | 1.38E-04 |
| GO:0009628 | BP | response to abiotic stimulus | 2 | 7 | 2.13E-04 |
| GO:0007601 | BP | visual perception | 2 | 4 | 3.92E-04 |
| GO:0050953 | BP | sensory perception of light stimulus | 2 | 4 | 3.92E-04 |
